# Plasma-Assisted Extraction of Polysaccharides from *Siegesbeckia orientalis* L.: Optimization, Purification, and Structural Characterization

**DOI:** 10.3390/polym18131568

**Published:** 2026-06-24

**Authors:** Yong-Hua Li, Li-Jie Zeng, Jin-Yun Wu, Jun Meng, Meng-Na Li, Jia-Yi Huang, Yan-Yan Huang, Feng-Song Liu

**Affiliations:** 1 Baiyunshan Hejigong Pharmaceutical Factory, Guangzhou Baiyunshan Pharmaceutical Holdings Co., Ltd., Guangzhou 510410, China; liyonghua@byshjg.com (Y.-H.L.); zenglijie@byshjg.com (L.-J.Z.); wujinyun@byshjg.com (J.-Y.W.); mengjun@byshjg.com (J.M.); 2 Guangdong Provincial Key Laboratory of Intelligent Food Manufacturing, School of Food Science and Engineering, Foshan University, Foshan 528225, China; mengnlimo@126.com (M.-N.L.); joyjiaaa@163.com (J.-Y.H.); 3 Guangxi Key Laboratory of Clean Pulp & Papermaking and Pollution Control, School of Light Industry and Food Engineering, Guangxi University, Nanning 530004, China

**Keywords:** *Siegesbeckia orientalis* L. polysaccharide, low-temperature plasma, structural characterization, rhamnogalacturonan-I (RG-I) pectin domain

## Abstract

Natural polysaccharides from *Siegesbeckia orientalis* L. have been reported to exhibit promising bioactivities. To enhance extraction efficiency, low-temperature plasma-assisted extraction was optimized for *S. orientalis* L. polysaccharides using single-factor experiments and response surface methodology. Column chromatography purified a homogeneous SIE-III fraction, followed by structural characterization. Optimal parameters were 80 kV discharge voltage, 153 Hz frequency, and 109 s treatment time, under which the polysaccharide yield reached 15.68%, significantly higher than that of the conventional hot water extraction method. Plasma treatment loosened the raw material’s surface, potentially facilitating polysaccharide release. SIE-III had a molecular weight of 20.831 kDa and comprised mainly galactose (51.7%), rhamnose (19.1%), arabinose (11.3%), and galacturonic acid (9.9%). It featured typical rhamnogalacturonan-I (RG-I) domains and a triple-helix conformation. Fourier transform infrared spectroscopy and nuclear magnetic resonance confirmed both α- and β- glycosidic linkages, and methylation analysis revealed a highly branched →3,4)-Galp-(1→ structure. This study provides an effective extraction method for plant polysaccharides and valuable insights into their potential applications in the food and other industries.

## 1. Introduction

*Siegesbeckia orientalis* L., a perennial Asteraceae herb, is rich in amino acids, polysaccharides, minerals, and various bioactive components, and possesses significant edible and traditional Chinese medicinal value [1,2]. Plant polysaccharides are natural polymers linked by glycosidic bonds. Recent studies have emphasized that plant polysaccharides exhibit multiple physiological activities, including antioxidant, anti-inflammatory, hypoglycemic, antibacterial and immunomodulatory effects [3,4,5,6,7]. With these unique functional properties, plant polysaccharides have become valuable raw materials in food processing, biomedicine and functional dietary supplements [8,9]. Studies have demonstrated that polysaccharides from *S. orientalis* L. exert prominent antioxidant, anti-inflammatory and immunomodulatory activities [10].

The biological properties of polysaccharides may be influenced by the integrity of their higher-order structures, while the selection of extraction methods directly affects both polysaccharide yield and structural integrity [11]. Currently, the major strategies for plant polysaccharide extraction include conventional hot-water extraction [12] and physical-assisted techniques, such as ultrasound-assisted extraction [13] and microwave-assisted extraction [14]. Although hot-water extraction is simple to operate and relatively inexpensive, it is generally associated with high extraction temperatures, prolonged processing times, and substantial energy consumption [15]. These conditions may induce polysaccharide backbone cleavage and glycosidic bond hydrolysis, potentially compromising structural integrity and biological properties [16]. Ultrasound- and microwave-assisted extraction can improve mass transfer efficiency and shorten extraction time; however, their broader application may still be limited by relatively high equipment costs, high sensitivity to process parameters, poor stability during scale-up, and structural heterogeneity caused by localized overheating [17,18]. Therefore, the development of novel extraction technologies with high efficiency, mild operating conditions, controllability, and good industrial scalability has become an important research direction in the field of plant polysaccharides.

Plasma-assisted extraction has emerged as a novel non-thermal physical extraction technology and has shown considerable potential for the efficient and mild extraction of plant polysaccharides because of its distinctive reactive-species-mediated effects and near-ambient operating conditions [19]. Its underlying mechanism is mainly attributed to the action of reactive oxygen and nitrogen species generated by plasma on the polysaccharide–lignin network within the cell wall. Through moderate oxidative depolymerization and microstructural remodeling of cell-wall components, cell-wall permeabilization and the release of bioactive compounds can be facilitated under mild conditions [20]. Dobrinčić et al. [21] applied non-thermal plasma pretreatment to the extraction of fucoidan from brown algae and found that the treatment effectively disrupted the cell wall, significantly improved extraction efficiency, and reduced extraction time. Nevertheless, plasma-assisted extraction still faces technical and economic challenges during industrial scale-up, including equipment investment, energy consumption control, and process parameter standardization, and its large-scale industrial application requires further development [22]. Compared with conventional hot-water extraction, plasma-assisted extraction can reduce the adverse effects of high temperatures and organic solvents on polysaccharide structures, thereby contributing to the preservation of higher-order conformations and functionally relevant structural features [23]. Therefore, plasma-assisted extraction not only provides an efficient and environmentally friendly extraction strategy but also represents a promising approach for the preparation of highly bioactive polysaccharides.

At present, systematic studies on the extraction process, structural characteristics and structure–activity relationship of *S. orientalis* L. polysaccharides are still limited. Considering the potential application value of *S. orientalis* L. polysaccharides in the fields of functional food and medicine, this study adopted a new plasma-assisted hot water extraction technology to prepare *S. orientalis* L. polysaccharides. The efficient extraction of polysaccharides was achieved by systematically optimizing the extraction process parameters, and the structural characteristics such as molecular weight, monosaccharide composition, glycosidic bond type and spatial conformation were characterized by high-performance gel permeation chromatography, ion chromatography, Fourier transform infrared spectroscopy and nuclear magnetic resonance. This study clarifies the regulatory mechanism of plasma treatment on the structural characteristics of *S. orientalis* L. polysaccharides, and provides a theoretical basis and technical support for the resource utilization and drug development of *S. orientalis* L. polysaccharides.

## 2. Materials and Methods

### 2.1. Reagents and Materials

*S. orientalis* L. was provided by Hejigong Pharmaceutical Factory of Guangdong Baiyunshan Group Co., Ltd. (Guangdong, China); D-glucose standard was purchased from Shanghai Aladdin Biochemical Technology Co., Ltd. (Shanghai, China); Dialysis bags (7000 Da, 44 mm) were purchased from Shanghai Yuanye Biotechnology Co., Ltd. (Shanghai, China); other reagents were of analytical grade/chromatographic grade.

### 2.2. S. orientalis L. Plasma-Assisted Extraction of Polysaccharides

The dried raw material of *S. orientalis* L. was crushed into powder and sieved through a 70-mesh sieve for later use. A certain amount (100 g) of *S. orientalis* L. powder from a single unified batch provided by the company was weighed, and the samples were pretreated using a CPCS-1-300 low-temperature plasma (Suzhou Yirun Food Technology Co., Ltd., Suzhou, China) under the following plasma conditions: interval time of 1500 ms, servo speed of 30 mm/s, electrode spacing of 25 mm, discharge voltage of 80 kV, discharge frequency of 110 Hz, and discharge time of 150 s. The treated *S. orientalis* L. samples were extracted in a water bath at 85 °C for 45 min at a solid–liquid ratio of 1:15 g/mL. The extract was vacuum-filtered through Whatman No. 1 filter paper, precipitated with ethanol overnight, and then centrifuged at 7000 rpm for 5 min. The precipitate was collected; ethanol was removed by rotary evaporation and freeze-dried to obtain the crude polysaccharides of *S. orientalis* L. The crude polysaccharide yield was defined as the percentage of freeze-dried crude polysaccharides relative to the initial mass of the raw *S. orientalis* L. powder and was calculated according to the following equation:

$$Polysaccharide\;yield(\%)=\frac{mass\;of\;freeze-dried\;crude\;polysaccharides(g)}{mass\;of\;raw\;S.orientalis\;L.\;powder(g)}\times 100\%$$

The total sugar content of the crude polysaccharides was determined using the phenol–sulfuric acid method. Briefly, 50 mg of crude *S. orientalis* L. polysaccharides was weighed and dissolved in 1 mL of pure water. In total, 200 μL of the sample was pipetted into a test tube separately, followed by the addition of 100 μL of pure water and 200 μL of 6% phenol, and then 1 mL of sulfuric acid was added. After shaking well and standing for 5 min, the mixture was heated in a water bath at 85 °C for 15 min, taken out and cooled to room temperature. A total of 100 μL of the mixture was pipetted to measure the absorbance at 490 nm. All measurements were performed in triplicate. The total sugar content was calculated by substituting into the standard curve.

### 2.3. Single-Factor and Response Surface Methodology Optimization Experiments

Based on preliminary experiments, single-factor experiments were carried out with five factors (discharge voltage, discharge frequency, discharge time, extraction time, and extraction temperature) at five levels, using the yield of *S. orientalis* L. polysaccharides as the index. The detailed factor and level design is presented as follows.

#### 2.3.1. Effect of Discharge Voltage on Polysaccharide Yield

A certain amount (20 g) of *S. orientalis* L. powder was weighed and pretreated under different discharge voltages (50 kV, 60 kV, 70 kV, 80 kV, and 90 kV) with a fixed discharge frequency of 130 Hz and a discharge time of 150 s. The samples were then extracted in a water bath at 85 °C for 45 min at a solid-liquid ratio of 1:15 g/mL.

#### 2.3.2. Effect of Discharge Frequency on Polysaccharide Yield

A certain amount (20 g) of *S. orientalis* L. powder was weighed and pretreated under different discharge frequencies (70 Hz, 100 Hz, 130 Hz, 160 Hz, and 190 Hz) with a fixed discharge voltage of 70 kV and a discharge time of 150 s. The samples were then extracted in a water bath at 85 °C for 45 min at a solid-liquid ratio of 1:15 g/mL.

#### 2.3.3. Effect of Discharge Time on Polysaccharide Yield

A certain amount (20 g) of *S. orientalis* L. powder was weighed and pretreated under different discharge times (90 s, 120 s, 150 s, 180 s, and 210 s) with a fixed discharge voltage of 70 kV and discharge frequency of 130 Hz. The samples were then extracted in a water bath at 85 °C for 45 min at a solid–liquid ratio of 1:15 g/mL.

#### 2.3.4. Effect of Extraction Time on Polysaccharide Yield

A certain amount (20 g) of *S. orientalis* L. powder was weighed and pretreated under fixed plasma conditions: discharge voltage of 70 kV, discharge frequency of 130 Hz, and discharge time of 150 s. The pretreated samples were then extracted in a water bath at 85 °C for different durations (25 min, 35 min, 45 min, 55 min, and 65 min) at a solid–liquid ratio of 1:15 g/mL.

#### 2.3.5. Effect of Extraction Temperature on Polysaccharide Yield

A certain amount (20 g) of *S. orientalis* L. powder was weighed and pretreated under fixed plasma conditions: discharge voltage of 70 kV, discharge frequency of 130 Hz, and discharge time of 150 s. The pretreated samples were then extracted in a water bath at different temperatures (75 °C, 80 °C, 85 °C, 90 °C, and 95 °C) for 45 min at a solid–liquid ratio of 1:15 g/mL.

### 2.4. Isolation of Crude Polysaccharides from S. orientalis L.

Pre-activated DEAE cellulose (100 g, Beijing Solarbio Science & Technology Co., Ltd., Beijing, China) was wet-packed into a column (Φ2.6 × 30 cm). The column was equilibrated with deionized water at a flow rate of 1.0 mL/min controlled by a peristaltic pump for 24 h before use. The freeze-dried crude polysaccharides were redissolved in deionized water to prepare a 10 mg/mL sample solution, which was loaded onto the column along the inner wall. The column was eluted sequentially with deionized water and NaCl solutions (0.1 mol/L, 0.3 mol/L, 0.5 mol/L) at a flow rate of 1.0 mL/min, with 10 mL fractions collected per tube. The total sugar content in each eluate was monitored using the phenol-sulfuric acid method, and an elution curve was plotted based on the tube number and absorbance values. Fractions corresponding to the same elution concentration and peak were pooled according to the elution curve, and purified fractions designated SIE-I, SIE-II, SIE-III, and SIE-IV were obtained after rotary evaporation, dialysis, and freeze-drying.

### 2.5. Purification of Graded Fractions of Crude S. orientalis L. Polysaccharides

Sephadex G-75 (100 g, Beijing Solarbio Science & Technology Co., Ltd., Beijing, China) was fully swollen according to the manufacturer’s instructions, and floating gel fragments were removed. Subsequently, the gel was wet-packed into a chromatographic column (Φ1.6 × 60 cm) and equilibrated with deionized water for three column volumes for later use. The preliminarily isolated SIE-III fraction from the previous separation was redissolved to prepare a sample solution of 10 mg/mL, and then filtered through a 0.45 μm aqueous microporous membrane before sample loading along the column wall. The column was eluted with 0.3 mol/L NaCl solution at a flow rate of 0.5 mL/min, and eluates were collected at 5 mL per tube. The total sugar content of each eluted fraction was determined by the phenol sulfuric acid method, and the elution curve was plotted with tube number versus absorbance. Fractions with identical peaks at the same elution concentration were pooled based on the elution curve. Finally, the purified SIE-III was obtained by rotary evaporation, dialysis and freeze-drying.

### 2.6. Physicochemical Determination of SIE-III

#### 2.6.1. Determination of Total Sugar Content

The total sugar content was determined with slight modifications according to the method described by Dubois et al. [24]. A series of glucose standard solutions (0, 0.1, 0.2, 0.5, 1.0, 2.0 mg/mL) was precisely prepared. Exactly 1.0 mL of each standard solution was transferred into a capped test tube, followed by the addition of 0.5 mL freshly prepared 6% phenol solution. Subsequently, 2.5 mL concentrated sulfuric acid (analytical grade, 98%) was added rapidly. The mixture was incubated in a water bath at 37 °C for 10 min, and then transferred to a 96-well microplate to measure the absorbance at 490 nm. The standard curve was established, and the regression equation was obtained as follows: y = 382.17x − 30.904 (R^2^ = 0.9995).

#### 2.6.2. Determination of Protein Content

The protein content in deproteinized *S. orientalis* L. polysaccharide solution was determined using a BCA protein assay kit(Beijing Solarbio Science & Technology Co., Ltd., Beijing, China). The principle is as follows: Peptide bonds in proteins can reduce Cu^2+^ to Cu^+^, and the generated Cu^+^ further reacts with BCA reagents to form a stable purple-blue complex. The maximum absorbance (OD_562_) was measured at 562 nm using a microplate reader. Within a certain concentration range, protein concentration exhibited a good linear relationship with OD_562_ value, and the protein concentration could be quantitatively calculated according to the absorbance. A standard curve was plotted with protein concentration as the abscissa and OD_562_ as the ordinate. The regression equation was y = 0.743x + 0.010, with a correlation coefficient of R^2^ = 0.996. The results indicated that protein concentration showed a good linear correlation with absorbance in the range of 0.0–1.0 mg/mL.

#### 2.6.3. Determination of Sulfate Content

The sulfate content of SIE-III was determined by the barium chloride–gelatin turbidimetric method with slight modifications according to Pei et al. [25]. An appropriate amount of SIE-III sample was hydrolyzed with hydrochloric acid to release sulfate groups. After cooling and dilution, the hydrolysate was mixed with trichloroacetic acid solution and BaCl_2_–gelatin reagent. The reaction mixture was allowed to stand at room temperature for 15 min to form a stable barium sulfate suspension. The absorbance was measured at 420 nm using a microplate reader. Potassium sulfate (K_2_SO_4_) was used to prepare a series of standard solutions for constructing the calibration curve. The sulfate content of SIE-III was calculated from the standard curve and expressed as a percentage of dry weight.

### 2.7. UV Spectral Scanning

The ultraviolet absorption spectrum of SIE-III (1 mg/mL) was scanned over the wavelength range of 200–600 nm using an ultraviolet spectrophotometer.

### 2.8. Determination of Microscopic Morphology

After gold sputtering for 120 s, the SIE-III sample was placed on a specimen stage. The surface morphological characteristics were observed by SEM (Quattro ESEM, Thermo Fisher Scientific, Waltham, MA, USA) at an accelerating voltage of 5 kV.

### 2.9. Molecular Weight Determination of SIE-III

The method described by Jiang et al. [26] was adopted with slight modifications. The molecular weight of SIE-III was determined using a high-performance liquid chromatography system (LC-10A, Shimadzu Corporation, Kyoto, Japan) equipped with a refractive index detector (RID-20A, Shimadzu) and a serial gel column (8 × 300 mm, BRT). The column temperature was maintained at 40 °C, and a 0.05 mol/L NaCl solution was used as the mobile phase at a flow rate of 0.7 mL/min. A total of 100 μL of dextran standard solution and SIE-III sample solution (5 mg/mL) were filtered through a 0.22 μm aqueous microporous membrane before injection for determination.

### 2.10. Determination of Monosaccharide Composition

The monosaccharide composition of SIE-III was determined with slight modifications according to the method of Wen et al. [27]. Exactly 5.0 mg of each monosaccharide standard was weighed, and 2 mL of 3 mol/L trifluoroacetic acid (TFA) was added, followed by hydrolysis at 120 °C for 3 h. After hydrolysis, TFA was removed by nitrogen blowing, and the residue was redissolved in 5 mL of ultrapure water to prepare individual monosaccharide standard stock solutions. The mixed standard working solution was further prepared for establishing the calibration curve. Additionally, 5.0 mg of the SIE-III sample was weighed and subjected to the same acid hydrolysis procedure. After TFA removal by nitrogen blowing, the sample was redissolved in 5 mL of ultrapure water. Subsequently, 50 µL of the redissolved solution was diluted with 950 µL of deionized water, centrifuged at 12,000 rpm for 5 min, and the supernatant was filtered through a 0.22 μm membrane filter prior to injection.

Chromatographic analysis was performed on a Dionex Carbopac™ PA20 column (3 × 150 mm) equipped with an electrochemical detector. The column temperature was set at 30 °C with a flow rate of 0.3 mL/min and an injection volume of 5 μL. The mobile phase consisted of water (A), 15 mmol/L NaOH (B), and 15 mmol/L NaOH containing 100 mmol/L NaOAc (C). Gradient elution was carried out according to the following program:0–18 min, A/B/C (98.8:1.2:0, V/V); 18–30 min, A/B/C (50:50:0, V/V); 30.1–46 min, A/B/C (0:0:100, V/V); 46.1–50 min, A/B/C (0:100:0, V/V); 50.1–80 min, A/B/C (98.8:1.2:0, V/V)for column re-equilibration.

### 2.11. Determination of Triple-Helix Structure

The helix–coil transition of triple-helical polysaccharides is strongly dependent on alkali concentration [28]. To explore the advanced conformation of SIE-III polysaccharide in solution, the Congo red assay was used to verify whether SIE-III possessed the ability to form a triple-helix structure. In the experimental group, 1 mL of 80 μmol/L Congo red solution was mixed with 2 mL of SIE-III solution at different concentrations (0–0.5 mg/mL), followed by the addition of different volumes of NaOH solution (1 mg/mL) within the NaOH concentration range of 0–0.5 mol/L. In the control group, SIE-III was replaced with pure water. After standing for 10 min at room temperature in the dark, the characteristic absorption spectra were scanned in the wavelength range of 190–600 nm using a UV–visible spectrophotometer.

### 2.12. Determination of Fourier Transform Infrared Microscopy Spectroscopy

The structural characterization of SIE-III was performed using a Fourier transform infrared microscope spectrometer (Vector 33, Bruker Optics, Ettlingen, Germany) over the wavenumber range of 600–4000 cm^−1^ with a resolution of 4 cm^−1^.

### 2.13. Determination of TG-DTG Thermal Stability

Approximately 3 mg of SIE-III was weighed and analyzed by a thermogravimetric analyzer (PerkinElmer STA 8000, PerkinElmer, Inc., Shelton, CT, USA). Nitrogen was used as the protective carrier gas at a flow rate of 30 mL/min. The TG and DTG measurements were performed from 30 °C to 900 °C at a heating rate of 10 °C/min.

### 2.14. XRD Measurement

The XRD measurement was carried out with slight modifications according to the method of Aramsangtienchai et al. [29]. The crystal characteristics of SIE-III were determined by a high-resolution X-ray diffractometer (D8 DISCOVER, Bruker, Billerica, MA, USA) with a Cu target (λ = 1.5418 Å). The test conditions were set as follows: accelerating voltage 40 kV, current 40 mA, scanning range 2θ = 5–80°, and scanning rate 0.5°/min.

### 2.15. Periodic Acid Oxidation and Smith Degradation

The experiment was performed with slight modifications based on the method described by Ren et al. [30]. Exactly 20.0 mg of SIE-III sample was accurately weighed and dissolved in 20 mL of 15 mmol/L sodium periodate (NaIO_4_) solution. An equal volume of NaIO_4_ solution without a sample was set as the blank control, and the reaction was carried out at 4 °C in the dark. To monitor the reaction kinetics, 0.1 mL of reaction solution was sampled at 0, 6, 24, 48, 72 and 96 h, respectively. After dilution and constant volume adjustment, the absorbance at 223 nm was determined until the value became stable. The total consumption of periodic acid was calculated according to the NaIO_4_ standard curve. After the reaction was completed, 5 mL of ethylene glycol was added to quench the residual periodate, and the generated formic acid was quantified by titration with 0.01 mol/L standard NaOH solution.

Monosaccharide standards were hydrolyzed to prepare a standard stock solution (A). Glycerol, ethylene glycol and erythritol standards were separately prepared to obtain a standard stock solution (B). Upon completion of periodic acid oxidation, ethylene glycol was added to terminate the residual sodium periodate. The reaction solution was purified by dialysis with a 3.5 kDa membrane and concentrated to 1 mL by rotary evaporation. Subsequently, 100 mg of NaBH_4_ was added for reduction at room temperature for 12 h. The reaction was terminated by adding 200 μL acetic acid, followed by dialysis and freeze-drying to obtain the reduced polysaccharide. Then, 2 mg of the reduced polysaccharide was hydrolyzed with 2 mL of 3 mol/L trifluoroacetic acid (TFA) at 110 °C for 3 h. After drying by nitrogen blowing, the residue was redissolved in water, then diluted, centrifuged and filtered. The monosaccharide composition of Smith degradation products was analyzed according to the chromatographic conditions described in Section 2.15.

Glycerol, ethylene glycol and erythritol were determined using a Dionex CarboPac MA1 column (4 mm × 250 mm) coupled with an electrochemical detector. The column temperature was maintained at 30 °C with a flow rate of 0.4 mL/min and an injection volume of 25 μL. Water (A) and 200 mmol/L NaOH solution (B) were used as mobile phases under isocratic elution: 0–30 min, A/B = 50:50 (V/V).

### 2.16. Methylation and GC-MS Analysis

The methylation procedure was performed with slight modifications according to the method of Zhou et al. [31]. Briefly, 60 mg of SIE-III was subjected to stepwise reduction using a uronic acid reducer under strictly controlled pH conditions. The sample was first activated and reacted at pH 4.6 for 3 h, and then the pH was adjusted to 6.8 for further reduction for 2 h. This procedure was repeated 3–5 times to ensure complete reduction. The product was purified with a 1 kDa dialysis bag and freeze-dried, and the reduction efficiency was verified by monosaccharide composition analysis.

Subsequently, 2–3 mg of reduced SIE-III was weighed and fully dissolved in 1 mL of anhydrous DMSO. Methylation reagent solution A (catalyst) and solution B (methylating reagent) were added sequentially using a methylation kit (BRT-JJH, Borui Saccharide, Yangzhou, China). The reaction system was placed in a constant-temperature water bath at 30 °C and magnetically stirred for 60 min. After the reaction, 2 mL of ultrapure water was added to quench the reaction. The methylated product was hydrolyzed with 2 mmol/L TFA at 100 °C for 90 min, followed by reduction with sodium borohydride for 8 h and acetylation with acetic anhydride for 1 h to obtain partially methylated alditol acetate derivatives. The derivatized products were purified by liquid–liquid extraction and finally separated and identified by GC-MS (Agilent GCMS 6890-5973, Agilent Technologies, Inc., Santa Clara, CA, USA).

GC-MS conditions were set as follows: an RXI-5 SIL MS capillary column (30 m × 0.25 mm × 0.25 μm) was used with helium as the carrier gas at a flow rate of 1.0 mL/min. The initial temperature was maintained at 140 °C for 2 min, then increased to 280 °C at a rate of 3 °C/min and held for 5 min. The inlet temperature and detector temperature were both set at 250 °C.

### 2.17. Nuclear Magnetic Resonance (NMR) Analysis

Exactly 50 mg of SIE-III was dissolved in 0.5 mL of D_2_O and subjected to three repeated freeze–thaw cycles to fully replace the active hydrogen in the sample. Finally, the deuterated sample was redissolved in 0.5 mL of D_2_O. One-dimensional NMR spectra were recorded at 25 °C using a Bruker 600 MHz nuclear magnetic resonance spectrometer.

### 2.18. Statistical Analysis

All data were analyzed using SPSS 27.0.1 (SPSS Inc., Chicago, IL, USA), and the results were presented as mean ± standard deviation with three independent replicates (n = 3). One-way analysis of variance (ANOVA) combined with the Tukey test was applied for intergroup comparisons. (*p* < 0.05). GraphPad Prism 10.2.3 (GraphPad Software, San Diego, CA, USA) and MestReNova 16.0.0 (Mestrelab Research S.L., Santiago de Compostela, Spain) were used for figure plotting.

## 3. Results and Discussion

### 3.1. Results of Single-Factor Experiments of S. orientalis L. Polysaccharides

**Figure 1:**
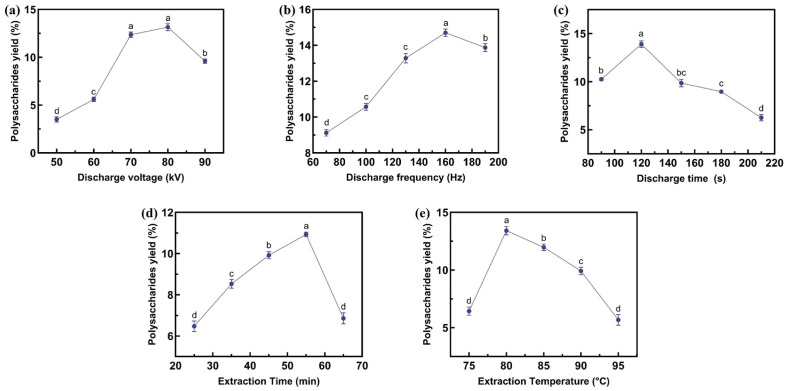
Single-factor experimental results. Effects of (**a**) discharge voltage, (**b**) discharge frequency, (**c**) discharge time, (**d**) extraction time, and (**e**) extraction temperature on the yield of *S. orientalis* L. polysaccharides. Note: Different lowercase letters indicate significant differences among different treatment groups (*p* < 0.05).

Plasma-assisted extraction can modulate the generation of reactive species by adjusting the discharge voltage, discharge power and treatment time [32]. Under controlled treatment conditions, the microstructure and surface properties of the plant matrix may be altered by these reactive species, thereby facilitating the release of polysaccharides [33]. Extraction time and extraction temperature mainly affect solvent penetration, polysaccharide diffusion behavior and structural stability. Therefore, single-factor experiments were first carried out on the above five key factors to clarify the influence rule and optimal range of each factor on polysaccharide yield to provide a basis for subsequent response surface optimization. The single-factor experimental results of the extraction process of *S. orientalis* L. polysaccharides are shown in Figure 1.

Figure 1a shows the effect of discharge voltage on the yield of *S. orientalis* L. polysaccharides. When the discharge voltage was increased from 50 kV to 80 kV, the yield of *S. orientalis* L. polysaccharides was increased most significantly, and the maximum polysaccharide yield was achieved at 80 kV. When the discharge voltage was further increased from 80 kV to 90 kV, the yield of *S. orientalis* L. polysaccharides decreased with the increase in discharge voltage. This trend indicated that insufficient plasma active particles were generated at low voltage, leading to low polysaccharide dissolution efficiency. Excessively high voltage might aggravate the oxidation effect of polysaccharides, thereby reducing the yield.

Figure 1b shows the effect of discharge power on the yield of *S. orientalis* L. polysaccharides. Within the discharge power of 160 Hz, the yield of *S. orientalis* L. polysaccharides was positively correlated with the discharge power. This was because the increase in discharge power could increase plasma density and energy, which strengthened the physical and chemical destruction of cell walls. However, after 160 Hz, further increasing the discharge power led to a decrease in polysaccharide yield, which might be caused by the breakage of polysaccharide chains induced by excessive energy input.

Figure 1c shows the effect of discharge time on the yield of *S. orientalis* L. polysaccharides. Within the discharge time of 120 s, the yield of *S. orientalis* L. polysaccharides was positively correlated with the discharge time. Appropriately prolonging the discharge time could ensure sufficient contact and reaction, thus improving polysaccharide dissolution. When the discharge time was increased from 120 s to 210 s, the yield of *S. orientalis* L. polysaccharides decreased with the extension of discharge time. This trend indicated that long-term exposure might lead to excessive accumulation of free radicals.

Figure 1d shows the effect of extraction time on the yield of *S. orientalis* L. polysaccharides. When the extraction time was extended from 25 min to 55 min, the yield of *S. orientalis* L. polysaccharides significantly increased and reached the peak at 55 min, followed by a decline. This change trend might be due to the fact that prolonging the extraction time in the early stage was conducive to the penetration of solvent and the establishment of mass transfer equilibrium, thereby promoting the full dissolution of polysaccharides from the plant matrix and their diffusion into the solvent; the decrease in polysaccharide yield in the later stage might be caused by polysaccharide hydrolysis induced by long-term thermal exposure.

Figure 1e shows the effect of extraction temperature on the yield of *S. orientalis* L. polysaccharides. As the extraction temperature was increased from 75 °C to 80 °C, the yield of *S. orientalis* L. polysaccharides showed an upward trend, indicating that within a certain temperature range, the increase in temperature could improve the solubility of the solvent and accelerate molecular movement, thereby promoting the dissolution of polysaccharides. When the extraction temperature exceeded 80 °C, the yield of *S. orientalis* L. polysaccharides showed a downward trend, which might be attributed to polysaccharide degradation, thereby reducing the yield.

### 3.2. Optimization Results of S. orientalis L. Polysaccharides by Response Surface Methodology

Single-factor experiments can only analyze the influence of a single variable, while the interaction among multiple factors and the optimal parameter combination cannot be revealed. Response surface methodology (RSM) has been widely applied to fit the nonlinear relationship between multiple factors and the response value (polysaccharide yield). A prediction model can be established to solve the optimal process parameters, and the significance and interaction intensity of various factors can also be evaluated [34]. Based on the single-factor experimental results of *S. orientalis* L. polysaccharides, three factors with three levels were selected for response surface optimization, including discharge time (90/120/150 s), discharge voltage (70/80/90 kV) and discharge power (130/160/190 Hz). Extraction time was fixed at 55 min and extraction temperature was set at 80 °C. The polysaccharide yield of *S. orientalis* L. was taken as the evaluation index. The experimental design was completed using Design Expert 13, as listed in Table 1. The detailed design matrix and results of the response surface methodology are presented in Table 2.

In this study, a process optimization model for plasma-assisted extraction of *S. orientalis* L. polysaccharides was established via response surface methodology. Based on the central composite design (Table 3), a quadratic multiple regression equation was obtained as follows:

Y = 15.63 − 1.06A − 1.11B − 1.39C + 0.8125AB + 0.6475AC + 0.8050BC − 2.57A^2^ − 2.33B^2^ − 2.12C^2^.

**Figure 2:**
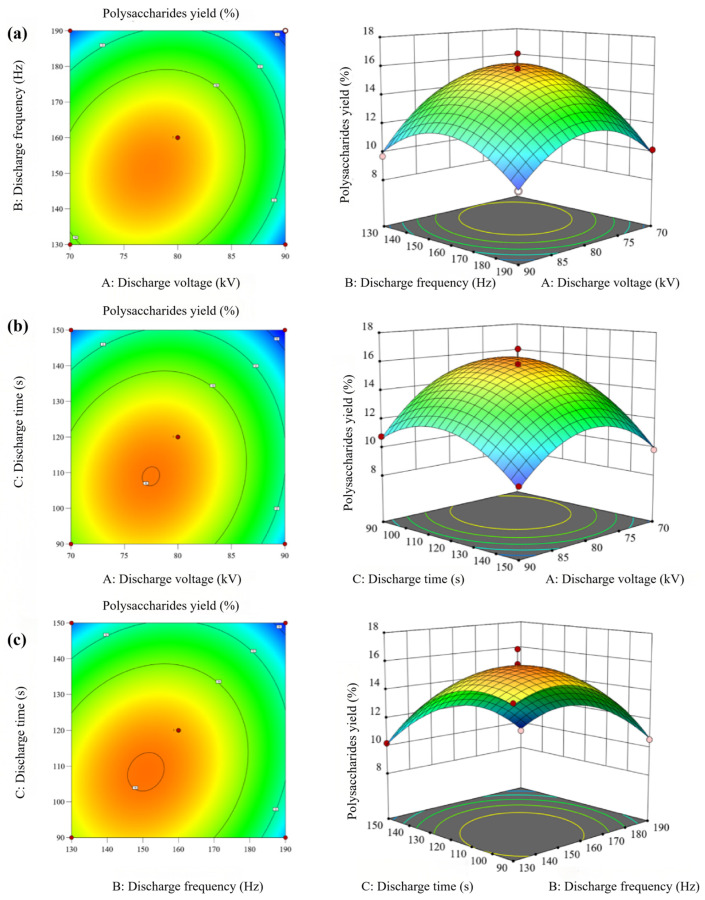
Simulation results of response surface methodology. (**a**) Discharge voltage and discharge frequency at a fixed discharge time of 120 s; (**b**) discharge voltage and discharge time at a fixed discharge frequency of 160 Hz; (**c**) discharge frequency and discharge time at a fixed discharge voltage of 80 kV. Note: The third factor was maintained at its center level during plotting. Note: Red solid circles represent the experimental design points, while the light pink circles indicate their projections onto the factor plane.

Excellent fitting degree and reliability were exhibited by the established model. The coefficient of variation (C.V.) was calculated to be 5.40%, indicating that 94.60% of the response variation could be explained by this model. The determination coefficient R^2^ of 0.9746 and adjusted determination coefficient Adj R^2^ of 0.9419 were both within reasonable ranges, which confirmed the strong predictive ability of the model. The lack-of-fit was not significant (*p* = 0.9186), indicating good model fitness. As revealed by the ANOVA results in Table 4, the most significant effect on polysaccharide yield was exerted by discharge time (C) (*p* = 0.0006), followed by discharge frequency (B) and discharge voltage (A). Highly significant differences were observed for all quadratic terms of the three factors (*p* < 0.001), suggesting the existence of obvious nonlinear relationships. According to the interaction analysis in Figure 2, the strongest interactive effect was found between discharge voltage and discharge frequency (AB), followed by discharge frequency and discharge time (BC), while the interaction between discharge voltage and discharge time (AC) was relatively weak.

The optimal process parameters were determined through model optimization: discharge voltage of 80 kV, discharge frequency of 153 Hz, and discharge time of 109 s. Verification experiments were carried out, and the actual polysaccharide yield was measured as (15.68 ± 0.21)%, with a deviation of only 2.3% from the predicted value of 16.05%. The reliability of the model was thus verified. Under these optimal conditions, an optimal balance between plasma-generated active species and plant cell wall action was achieved. Sufficient electric field strength was provided by voltage, adequate density of active particles was ensured by frequency, and sufficient reaction contact was guaranteed by time without causing excessive polysaccharide degradation. The findings of this study provide an important reference for industrial applications, and the correlation mechanism between plasma parameters and molecular characteristics of polysaccharides can be further explored in subsequent research.

### 3.3. Isolation, Purification and Physicochemical Characteristics of S. orientalis L. Polysaccharides

**Figure 3:**
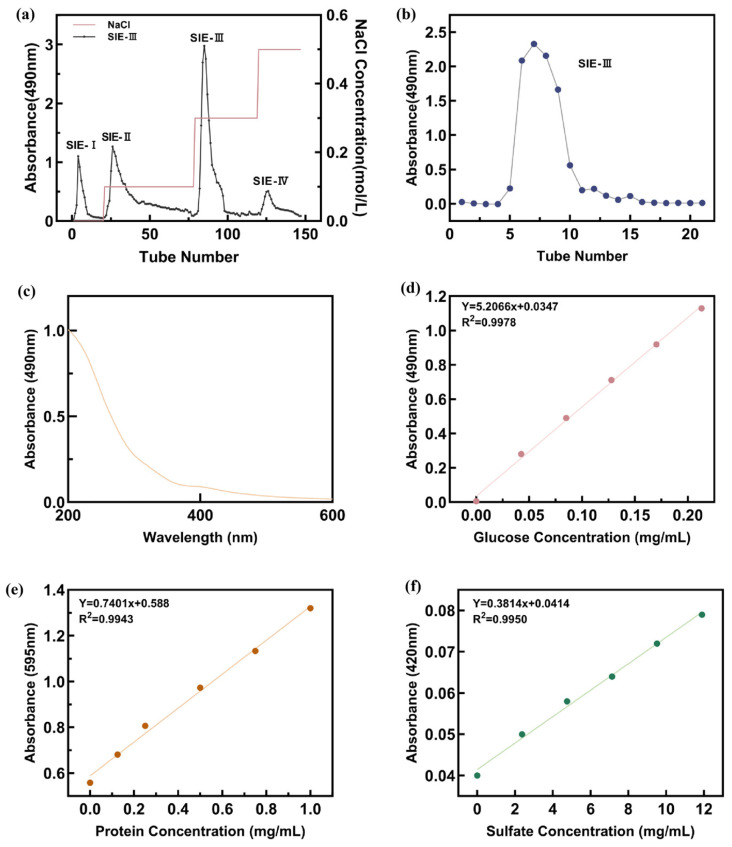
(**a**) Elution curve of DEAE cellulose chromatographic column; (**b**) elution curve of SIE-III on Sephadex G-75 gel column; (**c**) UV scanning spectrum of SIE-III polysaccharide; (**d**) glucose standard curve; (**e**) protein standard curve; (**f**) sulfate standard curve.

Purity and physicochemical properties are important quality indicators of polysaccharides, providing basic information on their chemical composition and suitability for subsequent structural characterization [35]. As shown in Figure 3a,b, crude polysaccharides extracted from *S. orientalis* L. were fractionated and purified by DEAE-52 ion-exchange chromatography. Gradient elution was carried out with 0–0.5 mol/L NaCl solution, and four polysaccharide fractions were obtained and designated as SIE-I, SIE-II, SIE-III and SIE-IV, respectively. A high and sharp characteristic peak was exhibited by SIE-III, indicating that this fraction possessed significantly higher polysaccharide content and better purity compared with the other fractions. Accordingly, SIE-III was selected for further purification. SIE-III was further separated by Sephadex G-75 gel filtration chromatography. The eluents corresponding to the symmetrical region of the main peak were collected, desalted by dialysis, and lyophilized to obtain a high-purity polysaccharide. Further physicochemical analyses (Figure 3c–f) indicated that the total sugar content of SIE-III was 90.61%, the protein content was only 0.27%, and the sulfate content was as low as 0.003%. In addition, no obvious absorption peaks were observed at 260 nm and 280 nm by ultraviolet spectral analysis, which demonstrated that the residues of nucleic acids and proteins in SIE-III were extremely low. The prepared SIE-III is suitable for subsequent structural elucidation and future bioactivity investigation.

### 3.4. Microscopic Morphology of SIE-III

**Figure 4:**
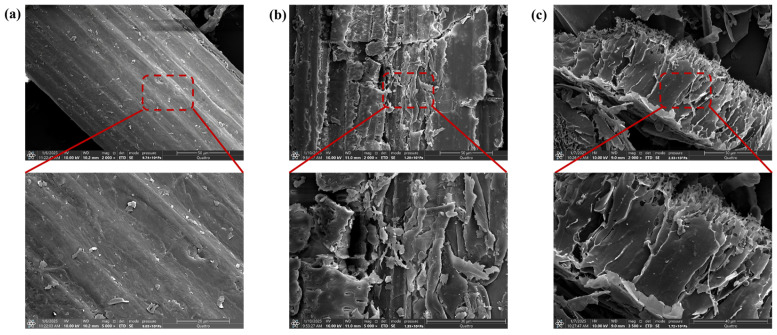
(**a**) Untreated *S. orientalis* L. raw material; (**b**) plasma-treated *S. orientalis* L. raw material; (**c**) microscopic morphology of SIE-III (2000×, 5000×).

Scanning electron microscopy (SEM) is one of the dominant techniques currently adopted to investigate the conformational characteristics of polysaccharides, which is attributed to its high resolution and magnification capability. Detailed surface images of samples can be acquired by collecting signals generated from the interaction between scanning electron beams and samples, thereby enabling the observation of surface morphology and microstructure [36]. Figure 4a,b shows the microscopic structure of *S. orientalis* L. raw materials before and after plasma treatment, respectively. A compact surface structure and closely arranged fibers were observed in the untreated raw materials. After plasma treatment, obvious physical etching and structural loosening were present on the material surface. Such changes may facilitate the deep penetration of extraction solvents and the outward diffusion of intracellular polysaccharides, laying a solid foundation for the efficient extraction of polysaccharides. As shown in Figure 4c, SIE-III exhibited an alternating structure of irregular sheets and networks with folds and pores distributed on the surface. This structure may increase the accessible surface area and facilitate interactions with water molecules, potentially contributing to its solubility and functional properties [37].

### 3.5. Molecular Weight and Homogeneity Analysis of SIE-III

**Figure 5:**
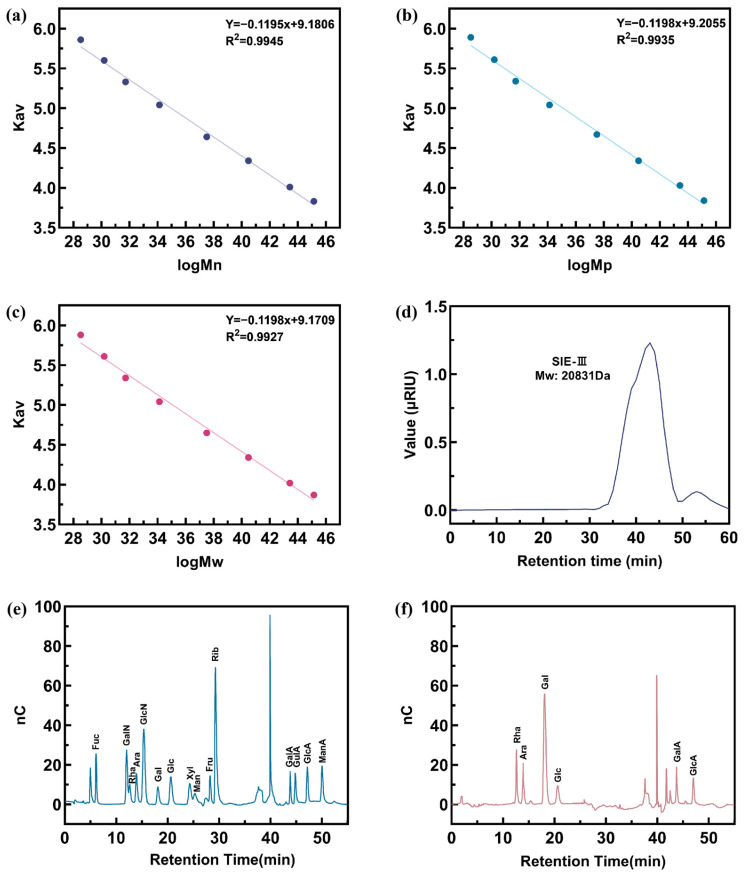
(**a**) logMn calibration curve; (**b**) logMp calibration curve; (**c**) logMw calibration curve; (**d**) molecular weight chromatogram of SIE-III; (**e**) chromatogram of monosaccharide standard mixture; (**f**) monosaccharide composition chromatogram of SIE-III.

Molecular weight and molecular-weight distribution are important structural parameters of polysaccharides, reflecting their molecular size and homogeneity [35,38]. Figure 5a–c display the calibration curves of number-average molecular weight (log Mn), peak molecular weight (log Mp) and weight-average molecular weight (log Mw) versus elution volume, respectively. The corresponding linear regression equations are Y = −0.1195x + 9.1806 (R^2^ = 0.9945), Y = −0.1198x + 9.2055 (R^2^ = 0.9935) and Y= −0.1198x + 9.1709 (R^2^ = 0.9927). The R^2^ values of all fitted curves were higher than 0.99, indicating a good linear relationship between the logarithm of molecular weight and elution volume. The separation characteristics of size-exclusion chromatography were well satisfied, and the reliability of molecular weight determination was verified. As observed from Figure 5d, the weight-average molecular weight (Mw) of SIE-III was determined to be 20.831 kDa. The distribution curve presented a symmetrical single peak with a narrow peak shape, demonstrating that SIE-III possessed a relatively homogeneous molecular weight distribution and high purity. This molecular weight characteristic was consistent with the molecular weight range of polysaccharides with remarkable biological activities reported in previous literature. Studies have indicated that the molecular weight of polysaccharides can substantially influence their solubility and molecular conformation, with relatively low or moderate molecular weights generally being more favorable for maintaining good solubility [39]. Previous studies have also suggested that such structural characteristics may be associated with antioxidant properties by facilitating free-radical scavenging through electron transfer and the chelation of catalytic metal ions [40]. Moreover, biological activities such as enzyme inhibition and cellular signal regulation can be realized via specific recognition with biomacromolecules [41]. In contrast, the biological activities of polysaccharides with higher molecular weight are often restricted due to sugar chain entanglement and active site masking [42].

### 3.6. Monosaccharide Composition Analysis

Monosaccharide composition is a fundamental structural characteristic of polysaccharides, providing important information on their constituent sugars, relative molar proportions, and potential backbone and side-chain features [38]. Polysaccharide types and domain structural features can be preliminarily inferred according to monosaccharide molar ratios. As indicated by the monosaccharide composition results in Figure 5e,f and Table 4, SIE-III is mainly composed of galactose (Gal), rhamnose (Rha), arabinose (Ara) and galacturonic acid (GalA), with a small amount of glucose (Glc) and glucuronic acid (GlcA). Such compositional characteristics suggest that SIE-III may be a pectic polysaccharide with a rhamnogalacturonan I (RG-I) structure [43]. As the predominant component, Gal implies that the backbone of SIE-III may be composed of galactan or rich in galactose side chains. Notably, the molar ratio of Rha to GalA is close to 2:1, which conforms to the typical structural features of RG-I polysaccharides. The presence of Ara further indicates the possible existence of arabinogalactan side-chain structures [44,45]. Compared with the polysaccharides reported in the literature, SIE-III exhibits unique compositional characteristics. Unlike GEP-U, a glucan dominated by Glc (98.77%) as reported by Zhou et al. [46], and MFP-3, a highly acidic polysaccharide rich in Rha (48.83%) as studied by Lei et al. [47], SIE-III is dominated by Gal and presents a complex structural feature containing both Rha and uronic acids. This specific monosaccharide composition may provide a structural basis for the potential biological properties. Previous studies have suggested that Gal-rich polysaccharides may be recognized and utilized by specific intestinal flora as a prebiotic, thereby potentially promoting the growth and metabolism of beneficial microorganisms [48]. Meanwhile, the highly branched side-chain network in the RG-I structure has been reported to provide diverse ligands for pattern recognition receptors on the surface of immune cells, potentially participating in the activation of related signaling pathways [49]. In addition, turonic acid residues and their carboxyl groups have been associated with antioxidant properties in certain polysaccharides, possibly through the chelation of transition metal ions and the interruption of free-radical chain reactions [50]. Rha and Ara have also been reported to participate in macrophage-related signaling pathways, including the TLR4/NF-κB pathway, in certain polysaccharide systems [51].

### 3.7. Triple Helix Structure Analysis

**Figure 6:**
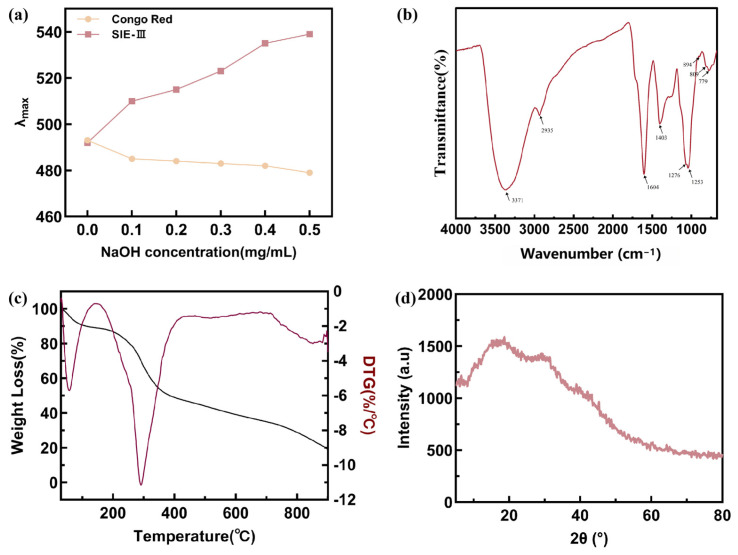
SIE-III polysaccharide. (**a**) Triple helix structure; (**b**) Fourier transform infrared spectrum; (**c**) thermal stability; (**d**) XRD pattern.

Higher-order conformation is an important structural characteristic of polysaccharides, and the Congo red assay is commonly used to preliminarily evaluate the presence of a triple-helix structure [52,53]. The Congo red method can be used to preliminarily determine the presence of a triple helix structure, which provides an important basis for evaluating the higher-order conformation of polysaccharides. As shown in Figure 6a, SIE-III exhibited a typical triple helix structure. Within the NaOH concentration range of 0–0.5 mol/L, a significant red shift was observed in the maximum absorption wavelength of the SIE-III–Congo red complex, indicating the presence of a triple helix structure in SIE-III.

### 3.8. Fourier Transform Infrared Spectroscopy Analysis

Fourier transform infrared spectroscopy (FTIR) serves as a core technique for rapidly identifying functional groups, judging glycosidic bond configurations, pyranose/furanose ring types and characteristic groups of polysaccharides. The structural type of polysaccharides can be preliminarily verified at the molecular level, providing supportive evidence for subsequent methylation and NMR analysis. As displayed in Figure 6b, SIE-III presented typical characteristic absorption spectra of polysaccharides. The broad and strong absorption peak at 3350 cm^−1^ was assigned to the O–H stretching vibration, and the weak absorption peak near 2900 cm^−1^ corresponded to the C–H stretching vibration of sugar rings [54]. The absorption band at 1650 cm^−1^ was attributed to the O–H bending vibration of bound water [55]. Monosaccharide composition analysis confirmed that SIE-III contained arabinose and galactose. Combined with branching and terminal sugar residues identified by methylation analysis, such as →5)-Araf-(1→ and →3,5)-Araf-(1→, the existence of complex arabinogalactan side chains in SIE-III was revealed. This structural feature was consistent with the absorption band formed by the stretching vibrations of C–O–C and C–OH in the side chains within the range of 1200–1000 cm^−1^ [56]. Notably, characteristic absorption peaks at 894 cm^−1^ and 779 cm^−1^ verified the presence of β-glycosidic bonds in SIE-III, while the absorption peak at 809 cm^−1^ corresponded to α-glycosidic bonds [57]. These results were not only consistent with the methylation data of →2)-Rhap and →4)-GalpA residues, but also in accordance with the conformational characteristics of polysaccharides reported by Yang et al. [58].

### 3.9. Thermal Stability Analysis

Thermal stability analysis (TG/DTG) reflects the thermal decomposition characteristics and heat resistance of polysaccharides under processing, sterilization and storage conditions, and acts as a key index for evaluating their industrial application feasibility in food and pharmaceutical fields [59]. The thermal degradation behavior of SIE-III was clearly divided into three characteristic stages by thermogravimetric analysis (Figure 6c). In the initial dehydration stage (30.9–209.9 °C), the weight loss (Δw1 = 14.10%) was mainly attributed to the desorption of physically adsorbed water and the volatilization of low-molecular-weight components [60]. The DTG inflection point at 55.63 °C corresponded to the glass transition temperature (Tg) of SIE-III, indicating the activation of molecular segment motion at this temperature. With the increase in temperature, thermal decomposition of SIE-III occurred at 209.91–555.87 °C, accompanied by a significant mass loss (Δw2 = 43.73%). An obvious degradation inflection point appeared at 291.39 °C, and the maximum weight loss rate was achieved within 270–310 °C. This stage was mainly involved in the cleavage of glycosidic bonds in the main chain of SIE-III and the ring-opening reaction of pyranose rings, generating CO_2_, H_2_O and other volatile small molecular products [61]. In the final carbonization stage (555.8–899.5 °C), the mass loss rate decreased significantly. The DTG inflection point at 851.01 °C marked the completion of the carbonization reaction. At this stage, the residues of SIE-III formed stable mineral ash through aromatization and condensation, accompanied by the release of a small amount of CO and CH_4_ [62]. The residual mass of SIE-III was 18.95%, demonstrating the relatively good thermal stability of the polysaccharide.

### 3.10. X-Ray Diffraction Analysis

X-ray diffraction (XRD) can be used to distinguish the crystalline or amorphous structure of polysaccharides. Amorphous structures have been reported to influence the physicochemical properties of polysaccharides, including their interactions with solvents. According to the analysis in Figure 6d, a broad diffuse diffraction peak was observed at 2θ of 10–30°, which was a typical characteristic of an amorphous structure. No obvious sharp crystal diffraction peaks were detected, indicating that the molecular chains of SIE-III mainly existed in a disordered amorphous state.

### 3.11. Analysis of Periodate Oxidation and Smith Degradation

**Figure 7:**
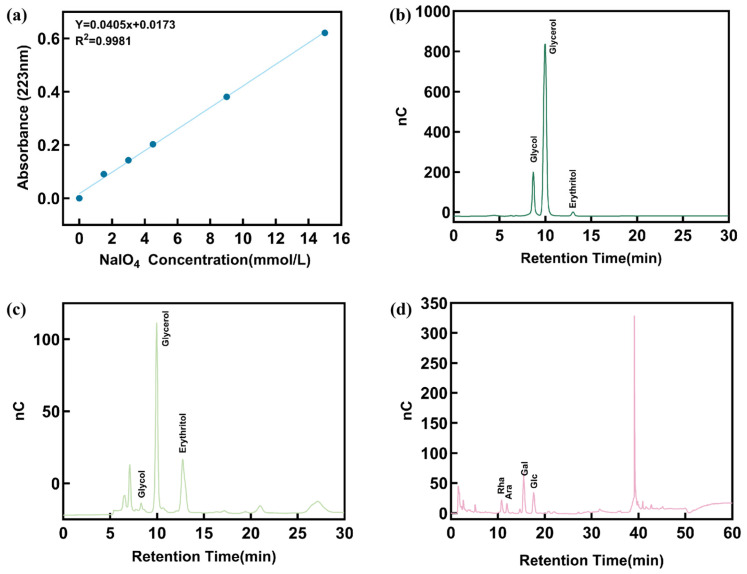
SIE-III. (**a**) Standard curve of NaIO_4_; (**b**) chromatogram of glycerol, hexanediol and erythritol standards; (**c**) chromatogram of glycerol and erythritol from Smith degradation products; (**d**) Full chromatogram of the Smith degradation products of SIE-III.

Periodic acid oxidation and Smith degradation are classical chemical methods for elucidating the glycosidic linkage modes of polysaccharides, which can be used to deduce the bonding characteristics of the main chains and side chains. To systematically analyze the glycosidic bond types of SIE-III, the total consumption of NaIO_4_ by 20 mg SIE-III was calculated to be 0.0722 mmol/L based on the NaIO_4_ standard curve (y = 0.0405x + 0.0173, R^2^ = 0.998), and the yield of formic acid was 0.02 mmol/L. These results indicated the presence of 1→ or 1→6 linkages in the molecule that could be oxidized by NaIO_4_ and release formic acid (HCOOH) (Figure 7a). Nevertheless, the molar ratio of NaIO_4_ to HCOOH was as high as 3.61, suggesting that besides 1→ and 1→6 linkages, other abundant linkage types existed in SIE-III. These linkages included 1→2, 1→2,6, 1→4 and 1→4,6 of pyranohexose, 1→ and 1→5 of furanopentose, as well as 1→2 and 1→4 of pyranodeoxyhexose. The oxidation law and corresponding degradation products of various glycosidic bonds are systematically summarized in Table 5.

To further accurately identify the linkage types cleaved by periodic acid oxidation, the original glycosidic bond types were inferred by identifying degradation products via GC-MS (Figure 7b–d). After Smith degradation, GlcA and GalA were completely undetectable in the monosaccharide composition, indicating that both uronic acid residues were located at oxidation-susceptible positions. Their linkage patterns were one or more of 1→, 1→2, 1→4, 1→6, 1→2,6 and 1→4,6, which were thoroughly degraded during subsequent acid hydrolysis. Meanwhile, the content of Ara decreased to 17.141 μg/mg, which was consistent with the reaction rule that furanopentose with 1→5 linkage was oxidized and degraded to produce glycerol and ethylene glycol. This result demonstrated that Ara mainly existed in the form of 1→5 linkage. Conversely, the relative proportions of Glc and Gal increased significantly, implying the existence of oxidation-resistant linkages such as 1→3, 1→2,3, 1→3,4, 1→3,6, 1→2,3,6 and 1→3,4,6. Erythritol detected in Smith degradation products is a characteristic product derived from the cleavage and reduction in pyranosidic bonds with 1→4 or 1→4,6 linkages. By contrast, glycerol may originate from multiple linkage types, including 1→, 1→2, 1→5, 1→6 and 1→2,6.

### 3.12. Methylation Analysis

Methylation analysis is an important method for elucidating the linkage patterns of sugar residues, branching positions, and the composition of main and side chains of polysaccharides, as well as for quantifying the molar ratio of each linked residue [63]. As shown in Table 6, SIE-III was predominantly composed of a highly branched →3,4)-Galp-(1→ domain with a molar ratio as high as 55.9%. This indicated that both the C3 and C4 positions of galactose residues were substituted, forming the core branching nodes of the SIE-III backbone. Owing to the absence of cis-vicinal dihydroxyl groups, this structural moiety was stable to periodic acid oxidation, which was consistent with the relatively low total consumption of periodate observed in the preceding periodic acid oxidation experiment. Meanwhile, SIE-III exhibited a high degree of terminal characteristics, and the total content of terminal sugar residues reached 14.5%. Among them, Araf-(1→ accounted for an absolute predominance of 8.7%, suggesting that Araf served as the major component at the outermost end of the side chains. Further structural analysis of the side chains revealed that →5)-Araf-(1→ acted as the main chain skeleton, with internal re-branching occurring via →3,5)-Araf-(1→, and the long chains were finally terminated by Araf-(1→. In addition, definite branching points including →4,6)-Galp-(1→ and →4,6)-Glcp-(1→ were identified from the methylation data, which acted as junction sites linking the side chains to the core skeleton of SIE-III. The presence of terminal Galp-(1→ and Rha-(1→ conformed to the typical structural features of rhamnogalacturonan I (RG-I), revealing that SIE-III may contain RG-I-type side chains composed of Rha and Gal residues [64].

### 3.13. NMR Analysis

Nuclear magnetic resonance (NMR) spectroscopy is one of the most important and powerful techniques for the structural identification of polysaccharides. Rapid and accurate structural analysis can be performed without destroying the sample conformation, thereby revealing the internal molecular arrangement and spatial conformation [65].

**Figure 8:**
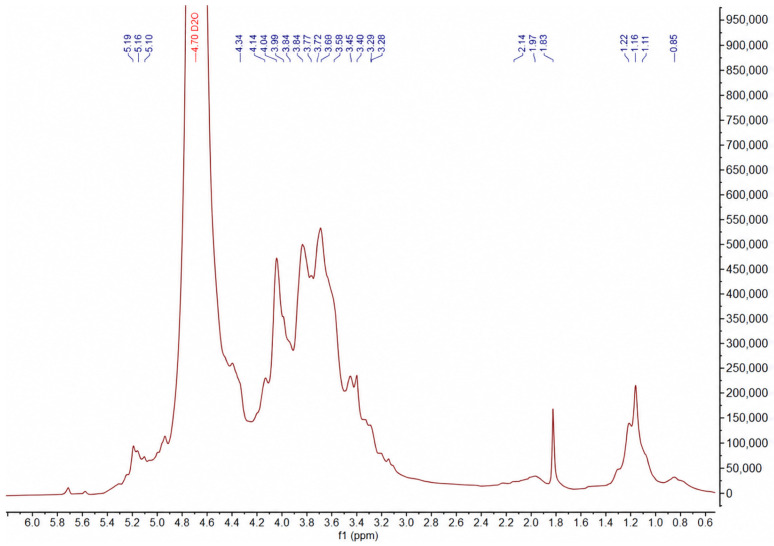
^1^H NMR spectrum of SIE-III.

In the ^1^H NMR spectrum(Figure 8), the anomeric hydrogen region at δ 4.5–5.5 ppm serves as the most critical range for identifying the configuration of polysaccharide glycosidic bonds [66]. Signals observed at δ 5.19 ppm, δ 5.16 ppm and δ 5.10 ppm were characteristic of α-glycosidic bonds, which were likely derived from residues such as Araf-(1→, Rhap-(1→ and →5)-Araf-(1→. By contrast, signals at δ 4.34 ppm and δ 4.14 ppm were assigned to β-glycosidic bonds, corresponding to Glcp-(1→ and Galp-(1→ residues [67]. The appearance of multiple signals in the anomeric region indicated that SIE-III was composed of various sugar residues with different configurations and linkage patterns. The broad and complex peak cluster at δ 3.0–4.5 ppm originated from the resonance absorption of C2–C6 hydrogen atoms on sugar rings [68]. The severe signal overlap in this region reflected abundant resonance of skeleton hydrogen atoms in both the main and side chains, which was typical of highly branched polysaccharide structures. This finding was highly consistent with the methylation results, further confirming that SIE-III possessed a →3,4)-Galp-based core skeleton enriched with arabinose side chains and rhamnose branching nodes. In addition, methyl hydrogen signals of Rhap C6 at δ 1.22 ppm and δ 1.11 ppm corresponded to the linkage modes of Rhap-(1→ and →2,4)-Rhap-(1→. Combined with the monosaccharide composition results, the existence of a typical rhamnogalacturonan I (RG-I) domain in SIE-III was conclusively verified [64].

**Figure 9:**
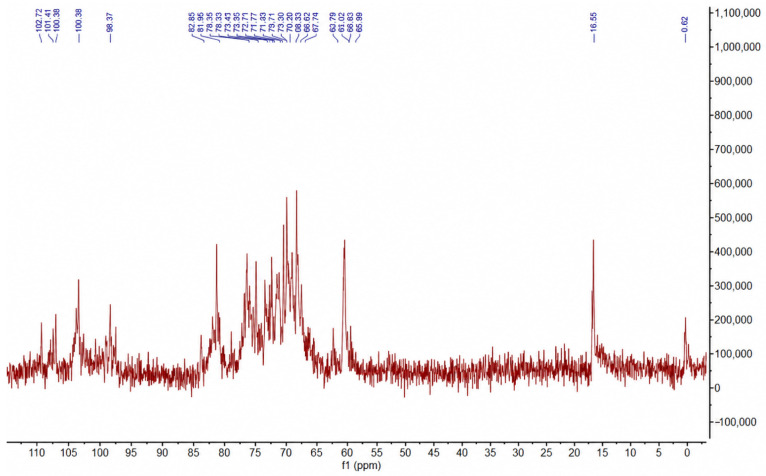
^13^C NMR spectrum of SIE-III.

In the ^13^C NMR spectrum(Figure 9), the anomeric carbon region at δ 95–110 ppm is regarded as the fingerprint region for polysaccharide structural elucidation [69]. The signal at δ 109.22 ppm was assigned to the anomeric carbon of α-furanose Araf, which was consistent with the methylation data of Araf-(1→ and →5)-Araf-(1→ [70]. The peak at δ 103.39 ppm corresponded to β-pyranose anomeric carbon, while δ 98.37 ppm was attributed to α-pyranose anomeric carbon, which agreed well with the corresponding α-anomeric hydrogen signals in the ^1^H NMR spectrum [64]. The broad and complicated peak cluster within δ 62–83 ppm was attributed to the resonance of C2–C6 carbons on sugar rings, reflecting the complex conformational characteristics of the polysaccharide skeleton [71]. Furthermore, the signal at δ 16.55 ppm was assigned to the C6 methyl carbon of rhamnose. Combined with the methyl hydrogen signals at δ 1.22 ppm and δ 1.11 ppm in the ^1^H NMR spectrum, the presence of an RG-I structural domain was further validated [72]. Previous studies have demonstrated that the RG-I domains may contribute to the functional properties of certain pectic polysaccharides [73]. Their side chains have been reported to interact with inflammation-related receptors, such as galectin-3 (Gal-3), and may be associated with an anti-inflammatory effect [74]. In addition, RG-I-containing pectic polysaccharides have been reported to alleviate renal injury, neuroinflammation, and cardiac inflammation in certain disease models [75]. These literature-based findings suggest a potential direction for future investigation of SIE-III. ^1^H and ^13^C chemical shift assignments for glycosyl residues of SIE-III are listed in Table 7.

## 4. Conclusions

The plasma-assisted extraction process significantly optimized the extraction efficiency of *S. orientalis* L. polysaccharides. The maximum polysaccharide yield of 15.68% was achieved under the optimal conditions: discharge voltage of 80 kV, discharge frequency of 153 Hz, discharge time of 109 s, extraction temperature of 80 °C, and extraction time of 55 min. A homogeneous polysaccharide fraction SIE-III was obtained after isolation and purification by DEAE-52 ion-exchange chromatography and Sephadex G-75 gel filtration chromatography. The total carbohydrate content of SIE-III reached 90.61%. SIE-III exhibited an amorphous structure, a triple helix conformation, and favorable thermal stability. Monosaccharide composition analysis indicated that SIE-III was mainly composed of galactose (51.7%), rhamnose (19.1%), arabinose (11.3%) and galacturonic acid (9.9%). The molar ratio of rhamnose to galacturonic acid was close to 2:1, suggesting that SIE-III belonged to a typical RG-I pectic polysaccharide. Methylation analysis and nuclear magnetic resonance spectroscopy further clarified that the core skeleton of SIE-III was highly branched →3,4)-Galp-(1→, and the side chains were rich in →5)-Araf-(1→ and terminal Araf residues, showing typical characteristics of the RG-I domain. The complex branched structure, especially the presence of the RG-I region, provides an important molecular basis for its potential biological activities. Meanwhile, this study also lays a theoretical foundation and provides a reference for the development and application of *S. orientalis* L. polysaccharides in functional foods and pharmaceutical fields.

## Figures and Tables

**Table 1:** Experimental design of response surface methodology.

Factors	Levels		
	−1	0	1
A-Discharge voltage (kV)	70	80	90
B-Discharge frequency (Hz)	130	160	190
C-Discharge time (s)	90	120	150

**Table 2:** Central composite design and results of response surface methodology.

Factors	A	B	C	Polysaccharide Yield (%)
1	90	190	120	9.31
2	90	130	120	9.68
3	80	160	120	14.67
4	70	160	90	13.85
5	80	160	120	15.83
6	80	160	120	15.63
7	70	130	120	13.76
8	90	160	150	9.32
9	80	130	90	14.62
10	80	190	150	9.35
11	80	190	90	10.56
12	70	190	120	10.14
13	80	160	120	15.13
14	80	160	120	16.88
15	80	130	150	10.19
16	90	160	90	10.77
17	70	160	150	9.81

**Table 3:** Analysis of variance (ANOVA) results of response surface methodology.

Source of Variance	Sum of Squares	Degree of Freedom	Mean Square	F-Value	Prob > F	Significance
Regression model	119.03	9	13.23	29.82	<0.0001	Significant
A-Discharge voltage	8.99	1	8.99	20.27	0.0028	Significant
B-Discharge frequency	9.88	1	9.88	22.27	0.0022	Significant
C-Discharge time	15.48	1	15.48	34.91	0.0006	Significant
AB	2.64	1	2.64	5.95	0.0448	Significant
AC	1.68	1	1.68	3.78	0.0929	Not significant
BC	2.59	1	2.59	5.84	0.0463	Significant
A^2^	27.9	1	27.9	62.89	<0.0001	Significant
B^2^	22.89	1	22.89	51.6	0.0002	Significant
C^2^	18.86	1	18.86	42.52	0.0003	Significant
Residual	3.1	7	0.4436			
Lack of fit	0.3308	3	0.1103	0.159	0.9186	Not significant
Pure error	2.77	4	0.6935			
Total	122.13	16				

**Table 4:** Monosaccharide composition and molar ratio of SIE-III.

Items	Rhamnose (Rha)	Arabinose (Ara)	Galactose (Gal)	Glucose (Glc)	Galacturonic Acid (GalA)	Glucuronic Acid (GlcA)
SIE-III	0.191	0.113	0.517	0.032	0.099	0.047

**Table 5:** Rules of periodic acid oxidation and Smith degradation for different glycosidic bonds.

Sugar Residue Type	Glycosidic Bond	Periodic Acid Oxidation Behavior	Smith Degradation Products
Pyranohexose	1→	Consumes 2 mol IO_4_^−^, produces 1 mol HCOOH	Glycerol, ethylene glycol
Pyranohexose	1→2	Consumes 1 mol IO_4_^−^, no HCOOH production	Glycerol
Pyranohexose	1→4	Consumes 1 mol IO_4_^−^, no HCOOH production	Erythritol
Pyranohexose	1→6	Consumes 2 mol IO_4_^−^, produces 1 mol HCOOH	Glycerol, ethylene glycol
Pyranohexose	1→2,6	Consumes 1 mol IO_4_^−^, no HCOOH production	Glycerol
Pyranohexose	1→4,6	Consumes 1 mol IO_4_^−^, no HCOOH	Erythritol
Furanopentose	1→	Consumes 1 mol IO_4_^−^, no HCOOH production	Glycerol, ethylene glycol
Furanopentose	1→5	Consumes 1 mol IO_4_^−^, no HCOOH production	Glycerol, ethylene glycol
Pyranodeoxyhexose	1→	Consumes 2 mol IO_4_^−^, produces 1 mol HCOOH	Ethylene glycol
Pyranodeoxyhexose	1→2	Consumes 1 mol IO_4_^−^, no HCOOH production	Glycerol
Pyranodeoxyhexose	1→4	Consumes 1 mol IO_4_^−^, no HCOOH production	Ethylene glycol

**Table 6:** Methylation analysis results of SIE-III.

RT	Methylated Sugar	Mass Fragments (*m*/*z*)	Molar Ratio	Type of Linkage
10.466	2,3,5-Me_3_-Araf	45, 71, 87, 101, 117, 129, 145, 161	8.7	Araf-(1→
11.730	2,3,4-Me_3_-Rhap	45, 59, 72, 89, 101, 115, 117, 131, 175	2.0	Rhap-(1→
13.881	2-Me_1_-Rhap	45, 87, 99, 113, 117, 129, 141, 159, 173	1.5	→3,4)-Rhap-(1→
15.119	2,3-Me_2_-Araf	45, 71, 87, 99, 101, 117, 129, 161, 189	6.4	→5)-Araf-(1→
15.357	2,3-Me_2_-Rhap	43, 59, 87, 101, 117, 129, 143, 161, 189	3.9	→4)-Rhap-(1→
16.824	2,3,4,6-Me_4_-Glcp	45, 71, 87, 101, 117, 129, 145, 161, 205	1.9	Glcp-(1→
17.741	2,3,4,6-Me_4_-Galp	45, 71, 87, 101, 117, 129, 145, 161, 205	1.9	Galp-(1→
18.379	2-Me_1_-Araf	45, 58, 85, 99, 117, 127, 159, 201	1.7	→3,5)-Araf-(1→
18.967	3-Me_1_-Rhap	45, 87, 101, 129, 145, 159, 189	4.5	→2,4)-Rhap-(1→
20.872	2,3,6-Me_3_-Galp	45, 87, 99, 101, 113, 117, 129, 131, 161, 173, 233	1.6	→4)-Galp-(1→
21.214	2,3,6-Me_3_-Glcp	45, 87, 99, 101, 113, 117, 129, 131, 161, 173, 233	3.4	→4)-Glcp-(1→
23.519	2,3,4-Me_3_-Galp	45, 87, 99, 101, 117, 129, 161, 189, 233	1.7	→6)-Galp-(1→
24.532	2,6-Me_2_-Galp	45, 87, 99, 117, 129, 143	55.9	→3,4)-Galp-(1→
26.408	2,3-Me_2_-Glcp	45, 71, 85, 87, 99, 101, 117, 127, 159, 161, 201, 261	1.7	→4,6)-Glcp-(1→
26.821	2,3-Me_2_-Galp	45, 71, 85, 87, 99, 101, 117, 127, 159, 161, 201, 261	3.3	→4,6)-Galp-(1→

**Table 7:** Chemical shifts (δ) for the resonances of glycosyl residues of SIE-III in ^1^H and ^13^C NMR spectra.

Residue		Chemical Shifts, δ (ppm)					
		H1/C1	H2/C2	H3/C3	H4/C4	H5/C5	H6/C6
A	t-α-L-Araf-(1→	5.19/109.22	4.14/81.5	3.93/76.5	4.04/83.9	3.77/61.7	—
B	→5)-α-L-Araf-(1→	5.16/107.47	4.14/81.5	3.93/76.5	4.04/83.9	3.84, 3.72/67.9	—
C	→3,5)-α-L-Araf-(1→	5.16/106.96	4.14/80.5	4.34/83.9	4.14/81.5	3.84, 3.72/67.9	—
D	α-L-Rhap-(1→	5.10/98.37	3.99/70.2	3.93/72.8	3.84/70.7	3.72/69.8	1.22/16.55
E	→4)-α-L-Rhap-(1→	5.10/98.37	3.99/70.2	3.93/72.8	4.14/78.9	3.72/69.8	1.16/16.55
F	→2,4)-α-L-Rhap-(1→	5.10/98.37	4.34/78.9	3.93/72.8	4.14/81.5	3.72/69.8	1.11/16.55
G	→4)-β-D-Galp-(1→	4.34/103.39	3.58/71.4	3.72/72.8	4.04/78.9	3.69/75.2	3.77/61.0
H	→3,4)-β-D-Galp-(1→	4.14/103.39 *	3.45/71.8	3.93/78.9	4.04/81.5	3.69/73.7	3.77/61.7
I	→4,6)-β-D-Galp-(1→	4.14/103.39 *	3.45/71.8	3.72/72.8	4.04/78.9	3.69/73.7	3.84, 3.72/67.9

Note: * indicates overlapped β-linked Galp/Glcp-type anomeric signals.

## Data Availability

The original contributions presented in this study are included in the article. Further inquiries can be directed to the corresponding authors.

## Abbreviations

The following abbreviations are used in this manuscript: Nrf2, Nuclear factor erythroid 2-related factor 2; NF-κB, Nuclear factor kappa-light-chain-enhancer of activated B cells; AGEs, Advanced Glycation End Products; D-Gal, D-Galactose; TLR4, Toll-like receptor 4; CP, Cold plasma; XRD, X-ray diffraction; TGA, Thermogravimetric analysis; DTG, Derivative thermogravimetry; NMR, Nuclear Magnetic Resonance; ROS, Reactive Oxygen Species; LPS, Lipopolysaccharide; MFI, Mean Fluorescence Intensity; ALT, Alanine Aminotransferase; AST, Aspartate Aminotransferase; TP, Total Protein.
